# Performance of wild-Serbian *Ganoderma lucidum* mycelium in treating synthetic sewage loading using batch bioreactor

**DOI:** 10.1038/s41598-019-52493-y

**Published:** 2019-11-06

**Authors:** Zarimah Mohd Hanafiah, Wan Hanna Melini Wan Mohtar, Hassimi Abu Hasan, Henriette Stokbro Jensen, Anita Klaus, Wan Abd Al Qadr Imad Wan-Mohtar

**Affiliations:** 1 Department of Civil and Structural Engineering, Faculty of Engineering and Built Environment, Universiti Kebangsaan Malaysia, 43600 UKM Bangi, Selangor, Malaysia; 2 Department of Chemical and Process Engineering, Faculty of Engineering and Built Environment, Universiti Kebangsaan Malaysia, 43600 UKM Bangi, Selangor, Malaysia; 3 Department of Chemical and Biological Engineering, The University of Sheffield, Sheffield, UK; 4 Institute for Food Technology and Biochemistry, Faculty of Agriculture, University of Belgrade, Nemanjina 6, Belgrade, 11080 Serbia; 5 Functional Omics and Bioprocess Development Laboratory, Institute of Biological Sciences, Faculty of Science, University of Malaya, Kuala Lumpur, 50603 Malaysia

**Keywords:** Fungi, Environmental sciences

## Abstract

The fluctuation of domestic wastewater characteristic inhibits the current conventional microbial-based treatment. The bioremediation fungi has received attention and reported to be an effective alternative to treat industrial wastewater. Similar efficient performance is envisaged for domestic wastewater whereby assessed performance of fungi for varying carbon-to-nitrogen ratios in domestic wastewater is crucial. Thus, the performance of pre-grown wild-Serbian *Ganoderma lucidum* mycelial pellets (GLMPs) was evaluated on four different synthetic domestic wastewaters under different conditions of initial pH (pH 4, 5, and 7) and chemical oxygen demand (COD) to nitrogen (COD/N) ratio of 3.6:1, 7.1:1, 14.2:1, and 17.8:1 (C3.6N1, C7.1N1, C14.2N1, and C17.8N1). The COD/N ratios with a constant concentration of ammonia–nitrogen (NH_3_–N) were chosen on the basis of the urban domestic wastewater characteristics sampled at the inlet basin of a sewage treatment plant (STP). The parameters of pH, COD, and NH_3_–N were measured periodically during the experiment. The wild-Serbian GLMPs efficiently removed the pollutants from the synthetic sewage. The COD/N ratio of C17.8N1 wastewater had the best COD and NH_3_–N removal, as compared to the lower COD/N ratio, and the shortest treatment time was obtained in an acidic environment at pH 4. The highest percentage for COD and NH_3_–N removal achieved was 96.0% and 93.2%, respectively. The results proved that the mycelium of GLMP has high potential in treating domestic wastewater, particularly at high organic content as a naturally sustainable bioremediation system.

## Introduction

A high fluctuation of sewage strength poses a limitation to an efficient treatment process in a sewage treatment plant (STP). Conventional STPs, which often use a biological treatment process, face challenges in ammonia removal in the nitrification and de-nitrification chambers for low sewage strength^1^. Ineffective sewage treatment consequently causes (point-source) pollution through the insufficiently treated sewage discharge into the waterways. The deteriorating water quality induces phenomena such as eutrophication or oxygen depletion in the water bodies, and potential hazards to human health (due to pathogens) are the pressing environmental issues related to a noncompliant sewage release^2^.

Ineffective ammonia removal may be caused by the low biodegradable organic content for the denitrifying bacteria to perform complete de-nitrification^3^. Low sewage strength can be attributed to the high infiltration of surface runoff into the sewage network^4^. Tropical countries may receive annual rainfall of up to 2500–3000 mm, whose high rainfall intensity may increase the infiltration rate^5^. Improper connections such as loose joints of, poor design of, and cracks in the sewer pipe walls of the sewer network assist in ensuring a high volume of runoff into the sewer systems. A conventional biological process treatment also requires additional cost because of the limitation of the nutrient concentration within the wastewater, and alternatives of nutrient addition are required to achieve the required nutrients for the treatment process. Nutrient balance in a biological treatment process is important to ensure optimum process performance. The most important nutrients are carbon (C), nitrogen (N), and phosphorous (P), whereby the maximum microbial activity in an aerobic wastewater treatment requires the C:N:P ratio to be maintained between 100:5:1 and 100:10:1^6^. The usual C/N ratio for collected domestic wastewater would be in the range of 12 to 16, which is categorised as high C/N ratio in this study. As such, the real wastewater is usually at high C/N ratio. Having said that, at places with high infiltration (such as tropical countries), the C/N ratio could be low (C/N <4), also in agreement by Mumtaz *et al*.^7^ for Malaysian wastewater and discharges.

Although bacteria-based wastewater treatment dominates the current process scenario, fungus-based treatment has received wide attention and proven to be an attractive alternative. Favourable outcomes of the bioremediation of fungi in the varying wastewater treatment include the use of the white rot fungus (WRF) strain *Trametes versicolor* in pharmaceuticals^8,9^ and WRF strains of *Funalia trogii*, *Phanerochaete chrysosporium*, *Pleurotus ostreatus*, *Pleurotus sajor-caju*, and *T*. *versicolor* in textile wastewater^10^. The WRF *T*. *versicolor* is the most promising fungus in the treatment of pharmaceutical compounds (PhACs), as it can degrade a wide range of PhACs because of its non-specific enzyme system properties^11^. Besides, the used immobilized WRF *T*. *versicolor* in real wastewater from food industries has shown promising results in the presence of extracellular enzyme activities^12^. WRF also plays an important role in the decolorisation of dyes from textile industries, where the fungal *Coriolus versicolor* strain achieves up to 97% efficiency of colour removal^13^. The WRF strains of *Funalia trogii*, *Phanerochaete chrysosporium*, *Pleurotus ostreatus*, *Pleurotus sajor-caju*, and *T*. *versicolor* were also successful in up to 90% removal of dyes^10^. Although WRF successfully studied in industrial wastewater, few studies reported on WRF treating the domestic wastewater. WRF strain *T*. *versicolor* was efficient in the removal of pharmaceutical compound (PCs) from the synthetic and real urban wastewater^14^. The application of the algal–fungal strain *Chlorella vulgaris and G*. *lucidum* in reducing COD, TN and TP from synthetic domestic wastewater was successfully performed by Xu *et al*.^15^.

WRF may degrade a diverse group of organic compounds besides lignin; therefore, the use of fungus-based bioremediation for wastewater has high potential. In particular, WRF can secrete a group of extracellular enzymes, including lignin peroxidases, manganese peroxidases, and laccases^16^. These enzymes allow the fungi to break down and utilise the organic substrate as an energy and nutrient source^17^. In contrast, in the conventional wastewater treatment, the type of bacteria is process dependent, and the process requires specific species of bacteria to produce a specific enzyme to degrade a specific target contaminant. The hyphal growth on the outer cell of the pellet fungi also provide resistance from the inhibitory compounds and pose favourable characteristics over bacteria^18^.

Utilising the advantageous fungi traits, this study evaluated the potential application of pre-grown wild-Serbian GLMP to the treatment of synthetic domestic wastewater under a sterile condition. The performance of the fungal wastewater treatment was assessed using various initial COD/N ratios emulating the fluctuating sewage loading into STP. Thus, this study was conducted in a small-scale batch bioreactor to determine the performance of the pellets in the domestic wastewater with respect to the pollutants normally found in sewage.

## Results and Discussion

The performance of wild-Serbian GLMP during the removal of COD and ammonia is described on the basis of the initial pH of the experiments. The results obtained at the initial pH of 7 will be discussed first, as it represents the common pH of the domestic wastewater. Results from the initial acidic conditions represent the optimal pH of WRF to remove effectively the pollutant. Thus, findings from the experimental setup at pH 5 and 4 will provide valuable information in high potential up-scaling fungi based wastewater treatment.

### Performance of fungi at initial pH of 7

**Figure 1:**
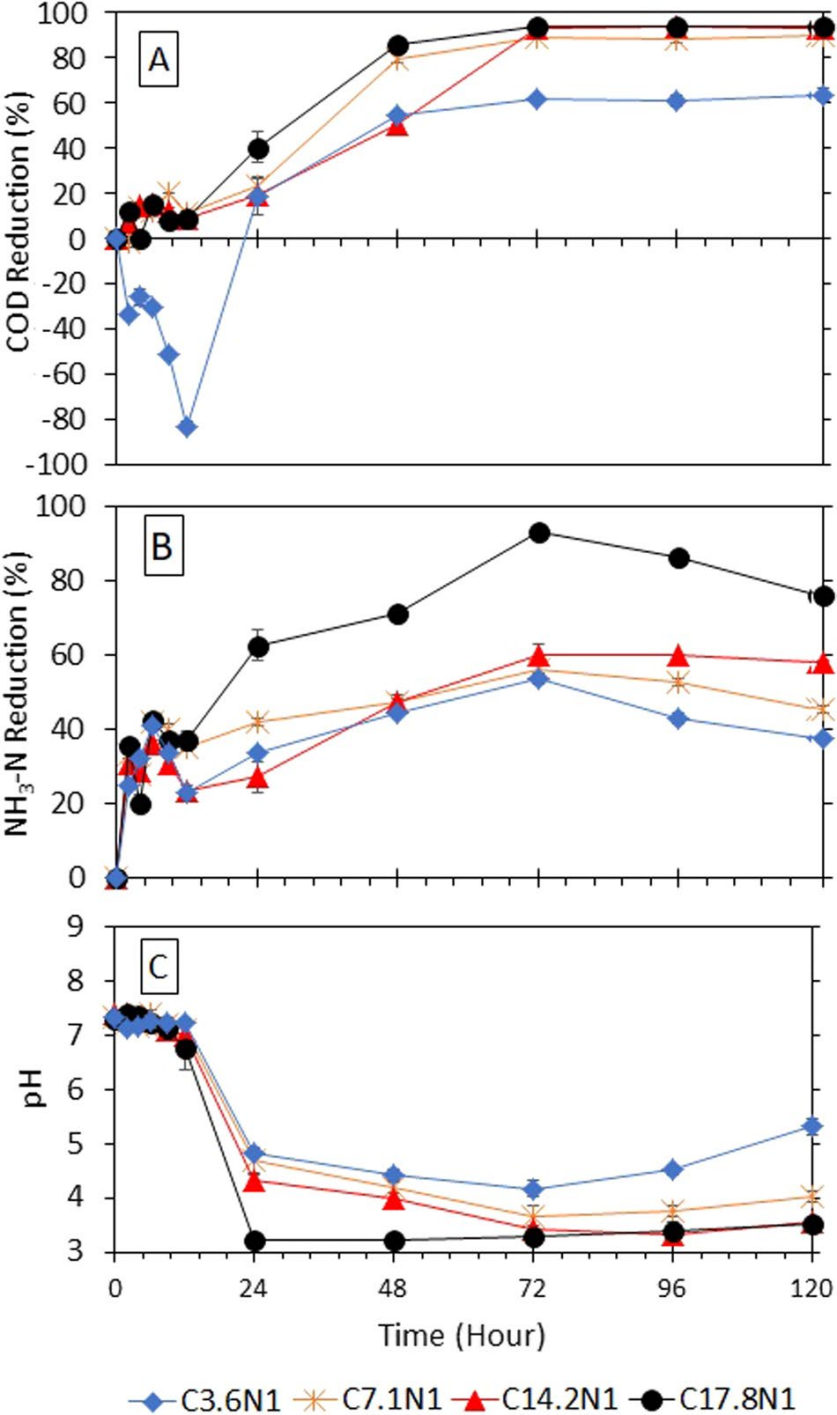
Percentage reductions of chemical oxygen demand (COD) (**A**) ammonia nitrogen (NH_3_–N) (**B**) and at influent of initial pH 7 (**C**).

The preliminary study of GLMP was conducted using the influent synthetic wastewater at the pH of 7.2 to 7.5, set at the mean pH value of the real sewage pH range, often measured to be between 6.5 and 8.5. In general, the results showed that the fungi successfully removed the pollutants from the synthetic wastewater and that the trends of pollutant removal were similar for all the tested COD/N ratios. The data shown in Fig. 1(C) indicate that within the first 12 h of treatment, the pH of the synthetic wastewater was maintained at its initial value, whereas the concentration of both COD and NH_3_–N (Fig. 1(A,B), respectively) showed a slightly increasing pattern.

The increasing pollutant concentration was believed to be influenced by the residual from the fungi growth medium during the pre-culturing process. The inoculum was pre-grown mycelial biomass for 10 days (stationary) fermentation whereas it is in the most active state. Yeast extract (1 g/L), Glucose (30 g/L), salts, and Ammonium chloride (source of Nitrogen, NH4Cl, 4 g/L) were the initial media used^19^ and has caused higher initial concentration of COD and nitrogen compounds. After 10 days, fungal residue has been detected by COD analysis due to catabolite repression or so-called “glucose effect^20^” which may give high pollutant concentration during the first hours of treatment at pH7.Beside that, the fungi can grow at initial hour as it consumed the pollutants as their carbon source, after the first hour the growth stopped due to unfavourable fungal growth conditions such as pH (*G*. *lucidum* grow best at pH 4^19,21^), cocktails of bacteria in the wastewater competing the same nutrients^22^, and natural *G*. *lucidum* survivability which caused pellet autolysis (cell death and cryptic growth)^23^ and samples were not 100% homogenous which lead to higher COD values in the initial treatment time.

After 24 h of treatment, the pH of the wastewater decreased for all the tested influents. The decreasing pH value was consistent with the increasing percentage removal of COD and NH_3_–N. The highest pollutant removal achieved followed the order of C17.8N1 > C14.2N1 > C7.1N1 > C3.6N1. In the case of the high COD/N ratio of C17.8N1, the percentage of COD and NH_3_–N removed was 40.3% and 62.7%, respectively, which was achieved after 24 h at a pH value of 3.24. The optimum condition was achieved at treatment time of 72 h with 93.8% and 93.2% of COD and NH_3_–N removal, respectively, at pH 3.28. After 72 h, the pH remained at 3.28, and the percentage removal of the pollutant showed no significant changes in COD over the next 48 h of treatment with 94.1% and 93.9% removal at 96 h and 120 h, respectively. However, the opposite trend was observed for ammonia where the performance of ammonia removal dropped to 86.4% at 96 h before continuously decreasing to 76.2% at 120 h. The decrease in the performance of ammonia removal was attributed to the cell death caused by the accumulation of metabolic waste^15^.

After a day, with the pH value at 4.82, the low COD/N ratio of C3.6N1 recorded a low percentage of COD removal with 18.4% of the organic content removed; however, a slightly higher NH_3_–N ammonia removal was observed (i.e. 33.9%). After three days, the pH was only reduced to 4.18, which was less than that in the case of the high COD/N ratio (C17.8N1) with a pH value of 3.28, and the performance of the fungi was not as expected as only 61.4% of COD and 53.6% of NH_3_–N were removed. The medium COD/N ratio of COD 213 (C7.1N1) and COD 427 (C14.2N1) showed moderate percentages of removal. For wastewater with C7.1N1, 23.0% COD removal and 42.1% NH_3_–N removal were recorded at pH 4.70 after 24 h. In contrast, an almost similar efficiency was observed for the C14.2N1 flask with 35.3% of COD and 36.5% of NH_3_–N removed at pH 4.25. However, when the treatment time was increased to 72 h, the treatment efficiency increased to the highest value of 88.5% COD removal and 56.1% NH_3_–N removal for C7.1N1 and 90.0% COD removal and 80.8% NH_3_–N removal for C14.2N1. Note that the pH value was logged at 3.65 and 3.32 for C7.1N1 and C14.2N1, respectively.

The data indicated that although fungi have the potential to treat organic and ammonia at the commonly measured pH of sewage, the treatment time required is rather long, reaching up to 48 h for all the COD/N ratios. GLMP took time to acclimatise to the ambient surroundings and only started to be efficient after 24 h, when the pH gradually decreased to below 4. This is quite consistent with the findings of a previous study on the removal of humic acid (monitored by colour reduction)^12^, wherein the acclimatisation of fungi *T*. *versicolor* was about 2 days and most of the colour was removed after 8 days. In another study, 24 h was needed for *P*. *chrysosporium* to be effective in removing phenol from synthetic oil refinery wastewater^24^. From here, fungi was adjusted the environmental pH for the fungi to utilised the pollutant and lead to a reduction in the pollutant concentration. The acidic environment obviously favoured the fungi^25^, who reported that effective fungal treatment was found within the pH range of 4.5.

### Performance of fungi at initial pH of 5

**Figure 2:**
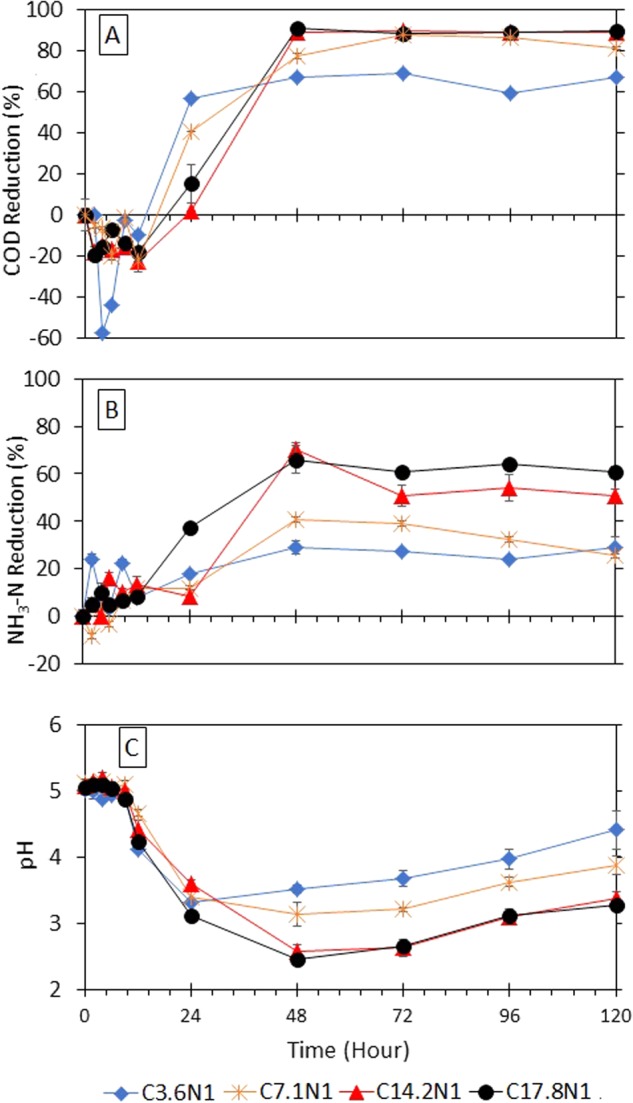
Percentage reductions of chemical oxygen demand (COD) (**A**) ammonia nitrogen (NH_3_–N) (**B**) and at influent of initial pH 5 (**C**).

Previous experiments (with initial pH of 7) have shown that GLMP took approximately 48 h to stabilise and acclimatise. To further investigate the ability of GLMP to self-modify its environmental pH and a possible reduction of treatment time, the initial pH of 5 in the reactor was introduced to the GLMP. The changes in the pH values within the reactor with an initial pH of 5 are plotted in Fig. 2(C). In general, the pH is visibly consistent from the kick-off until *t* = 6 h before it started to decrease between 4.77 and 4.13 at *t* = 12 h. During the acclimatisation period, the COD concentration increased (which describes the negative COD reduction in Fig. 2(A)) due to as previously discussed. However, the concentration of NH_3_–N remained consistent throughout the acclimatisation period for all the COD/N ratios (Fig. 2(B)). The data visibly showed that there was a time lag when the GLMP started to degrade NH_3_–N (with a notable exception of the highest COD/N ratio).

After 12 h of treatment, the pH started to decrease for all the considered ranges of the COD/N ratio discussed in this study. Corresponding to the decreasing pH at *t* = 24 h, the percentage removal of COD began to hit positive numbers. The decreasing trend of pH was consistent with the increasing percentage removal of COD and NH_3_–N. Similar findings (to those of the pH 7 experiments) were obtained where the highest pollutant removal was achieved for the reactor with the highest COD/N ratio (C17.8N1) and was sequentially followed by those for C14.2N1, C7.1N1, and C3.6N1. In the case of the highest COD/N ratio of C17.8N1, the percentages of COD removal and NH_3_–N removal were 15.3% and 37.3%, respectively, after 24 h of treatment at the recorded pH of 3.13. The maximum reduction was achieved at *t* = 48 h with 91.0% and 61.1% removal of COD and NH_3_–N, respectively at a much lower pH value of 2.47. After 48 h, the percentage removal of the pollutants reached its maximum potential with a steady pattern over the next 72 h of the treatment (88.6% COD removal and 61.0% NH_3_–N removal at 72 h, 89.1% COD removal and 64.4% NH_3_–N removal at 96 h, and 89.6% COD removal and 61.0% NH_3_–N removal at 120 h).

In contrast, the lowest COD/N ratio (C3.6N1) recorded 57.0% and 17.7% of COD and NH_3_–N removal, respectively, at *t* = 24 h with a measured pH of 3.33. On the basis of these data, the COD reduction followed a consistent pattern rather early after 24 h of treatment (with a maximum value of 67.0% at the end of the experiment). The removal of NH_3_–N was evidently lower than that at the other COD/N ratios and only reached a maximum value of 29.0% at *t* = 48 h. The medium wastewater strength of COD 200 mg/L (C7.1N1) and COD 400 mg/L (C14.2N1) showed a moderate percentage removal, particularly for NH_3_–N. After 24 h of treatment, 40.6% of COD and 11.9% of NH_3_–N were removed for C7.1N1, while a much lower percentage of 2.0% and 8.2% for COD and NH_3_–N removal, respectively, was observed for C14.2N1. Both the reactors recorded decreasing pH values within the range of 3.41 to 3.60 when increasing treatment time to 72 h increased the percentage of COD removal, where both the medium wastewater strengths recorded 87.9% and 89.7% for C7.1N1 and C14.2N1, respectively. Note that the C14.2N1 reactor produced 89.36% removal at *t* = 48 h, much earlier than when the C7.1N1 reactor had a lower percentage of removal (at *t* = 72 h).

The percentage of NH_3_–N removal was only 40.7% and 70.5% for C7.1N1 and C14.2N1, respectively, even though the pH was significantly reduced even to less than 3 (at 2.6) at *t* = 48 h. In this pH 2 environment, the fungi were believed to be in a non-detected growth activity phase^26^. In general, the potential of GLMP to reduce the NH_3_–N concentration was visibly much lower than that in the experiments with initial pH 7 for all the COD/N ratios. In the first 24 h of treatment, the percentage of removal was low, probably because the surrounding pH in the reactor was yet to achieve its desired optimum value.

### Performance of fungi at initial pH of 4

Another experiment was conducted to investigate the effect of the initial pH on the performance of GLMP at the lowest pH value of 4. We acknowledged that a more acidic environment at pH 4 was completely undesirable for possible large-scale applications, but we wanted to investigate the possibility of reducing the treatment time if the initial pH in the reactor was similar to the optimised pH of the GLMP growth. Our hypothesis, however, was proven to be inaccurate, as the GLMP still required 12 h to acclimatise with a visible reduction only observed at *t* = 24 h.

**Figure 3:**
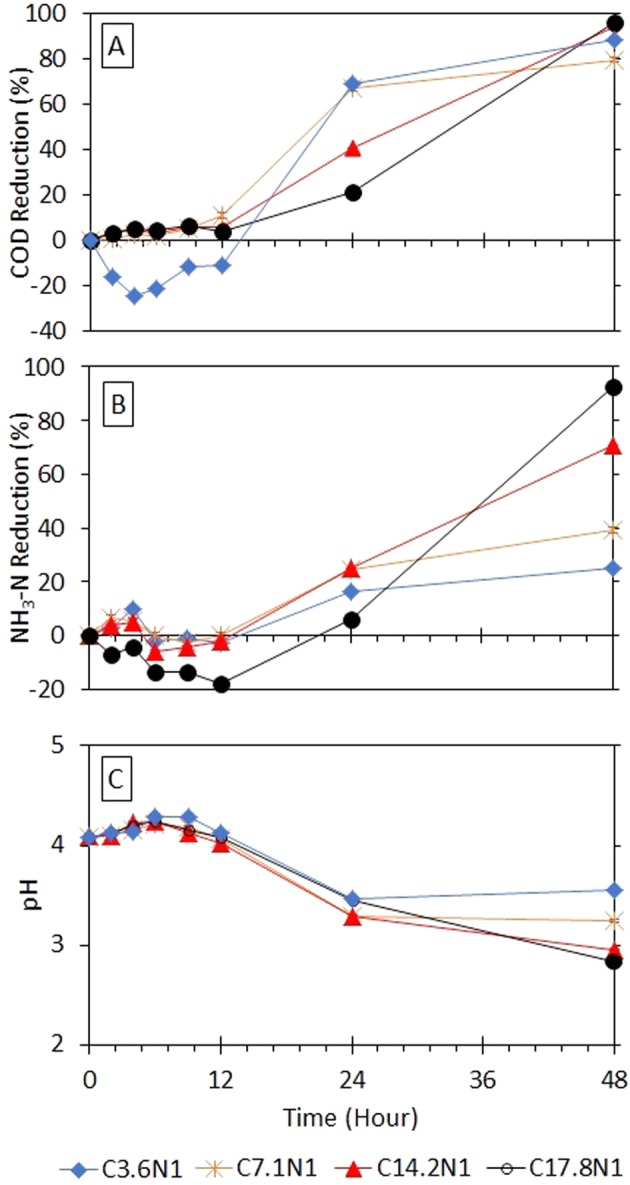
Percentage reductions of chemical oxygen demand (COD) (**A**) ammonia nitrogen (NH_3_–N) (**B**) and at influent of initial pH 4 (**C**).

The recorded monitoring pH in the reactor is shown in Fig. 3(C), with a slight increase in pH visible at *t* = 6 h, before it started to decrease to the range of 3.29 to 3.47 at *t* = 24 h. After 12 h of treatment, a fast decrease in the pH value was observed for all the COD/N ratios discussed in this study. The initial pH of 4 provided an acidic environment to the fungi to adapt to, and their response to induce the changes to a preferred pH value was much faster than that of pH 7. The rapid decrease in pH was consistent with the fast increasing percentage removal of COD and NH_3_–N. At the end of the experiment at *t* = 48 h, the lowest pH was measured for the C17.8N1 reactor, and the highest was for C3.6N1. Note that the duration of the experiment was shortened to 48 h, as the pH value was below 3. The rapid decrease in pH was consistent with the fast increasing percentage removal of COD and NH_3_–N.

The initial pH for the treatment obviously played no important role in accelerating the GLMP reaching its phase for the fungi to intake the pollutants. Interestingly here, the lowest COD/N ratio reactors improved its capability of reducing COD up to 79.7% and 88.2% for C7.1N1 and C3.6N1, respectively, at *t* = 48 h, as shown in Fig. 3(A). A significant increase in the COD removal was observed within 36 h of treatment for all the reactors. The trend in this experimental setup differed from that in the profile shown in the previous initial pH experiments, where a fast organic removal with 68.87% and 66.97% for the C3.6N1 and C7.1N1 reactors, respectively, was visible after 24 h of treatment, much faster than that in the case of the higher COD/N ratio wastewaters. However, both C3.6N1 and C7.1N1 were not able to achieve a similar percentage removal as shown in the C17.8N1 and C14.2N1 reactors at the end of the experiment at 96.0% and 94.1%, respectively. Interestingly, the reduction of NH_3_–N followed suit with the highest percentage recorded at 92.4% for C17.8N1 (Fig. 3(B)). According to the results obtained in the pH 7 and 5 experiments, the highest COD/N ratio (at C17.8N1) showed the most efficient percentage pollutant removal and was the fastest the considered COD/N ratios. High COD/N ratios in the wastewater can be considered mimicking the ligninolytic conditions (representing the real condition as wood has high C/N ratio). This scenario leads to increasing fungal production of lignin modifying enzymes^10,27^ and consequently increased the consumption of nutrient. In this experiment, high percentage of ammonia removal by the fungal pellets was observed.

Although an excellent removal of COD was observed for the C3.6N1 and C7.1N1 reactors, a contrasting profile of the NH_3_–N reduction was noticed. After 24 h of treatment, only 16.4% and 24.2% of NH_3_–N removal were calculated for C3.6N1 and C7.1N1, respectively, even though the pH in these reactors fell into the range of 3.29 to 3.47. A rather disappointing final NH_3_–N reduction was obtained for both the reactors where only 25.3% and 39.1% of the removal was achieved for C3.6N1 and C7.1N1, respectively. The medium COD/N ratio of C14.2N1 showed a better percentage removal where after 24 h: 40.6% COD removal and 25.1% NH_3_–N removal. Although the COD removal may be within the same range as that of the C17.8N1 reactor, the C14.2N1 wastewater achieved a much lower NH_3_–N reduction at 70.9%.

Although the performance of GLMP in reducing COD and NH_3_–N varied and can be considered carbon-dependent, the huge potential of utilising GLMP even at low wastewater strength (i.e. a low COD/N ratio) was predicted. Based on the results of the highest percentage of both COD and NH_3_–N removal with the shortest treatment time, the best condition of GLMP-based wastewater treatment was high COD at an initial pH of 4. The best condition of pH 4 in the pure culture of GLMP is comparable to the findings by Xu *et al*.^15^ which discussing the application of mixed algal-fungal strain (palletisation between *C*. *vulgaris* and *G*. *lucidum 5*.*765*) to treat synthetic domestic wastewater with different C/N ratio influents. The experiment was presented as low (100 mg/L), medium (200 mg/L), and high (400 mg/L) levels of COD (where the TN/TP level was fixed at medium strength). Wastewater strength at corresponding C/N ratios of C2.5N1-COD100, C5N1-COD200 and C10N1-COD400 was tested in a photobioreactor, set up in batch experiment for a much longer treatment time at 10 days.

The optimal condition achieved for the algal–fungal culture was for the C/N ratio C5N1 with a percentage removal for COD and total nitrogen (TN) of 74.41% and 79.35%. With respect to the C/N ratio, the C2.5N1 and C10N1 treatments had a significantly lower percentage removal with COD (74.79%) and TN (75.08%) for C2.5N1 and COD (71.60%) and TN (75.54%) for C10N1. The optimal pollution removal efficiency was achieved for treatment using the algal–fungal culture on day 8 with the pH of the synthetic wastewater consistent with the pH in the influent with the range of 6.0–7.5. The pure culture of *G*. *lucidum* showed advantages in the self-immobilisation of the pellet system and the long-activity maintenance of the secretion of bioactive metabolites^28^.

Our experimental work indicated that the culture of *G*. *lucidum* in the treatment of synthetic wastewater had a higher potential than the mixed culture. The pre-culture of the preparation of mycelium pellets for the pure fungus required 9–11 days as compared to the culturing condition for the algal–fungal culture, which required 14 days. Moreover, a higher percentage removal of organic pollutants, particularly COD, of about 96.0% was achieved as compared to 74.4% for the mix culture condition. The treatment with the pure culture of *G*. *lucidum* also had a short treatment time: under the optimal condition, the same results were achieved at 48 h of treatment under the initial acidic condition as those achieved after 8 days under the algal–fungal culture condition.

### Morphological observation

Like any other medicinal mushrooms, the *Ganoderma* species feed by the absorption of nutrients from their growth surroundings, such as synthetic nitrogen (N)^23^, ammonia nitrogen (NH_3_–N)^29^, atmospheric N^30^, and industrial dyes^31^. In a wastewater treatment application, adsorption has long been used for NH_3_–N removal^32^ using costly non-biological materials, and is insufficient to meet the current market demand. The use of biological materials such as medicinal mushrooms offers a better alternative as the fungal source can be produced in bulk, using a cheap and safe process, and to a consistent quality^33^.

**Figure 4:**
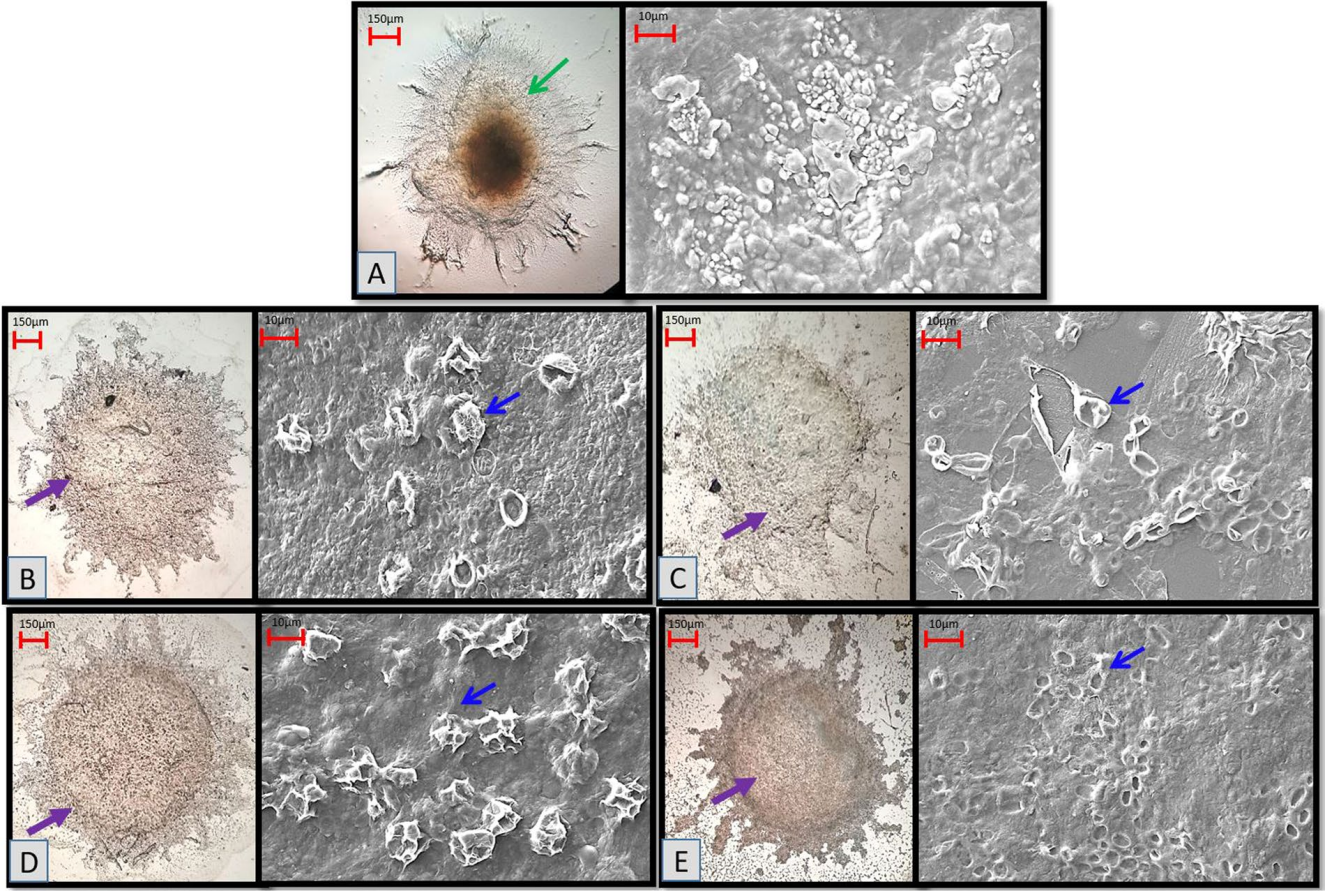
Wild-Serbian *G*. *lucidum* mycelial pellets (GLMPs) treated with synthetic wastewater in initial pH 4 at four-fold magnification using light microscope (LM-*left picture*) and variable pressure scanning electron microscope (VPSEM-*right picture*). Control: untreated GLMP (**A**), GLMP treatment in C3.6N1 (**B**), GLMP treatment in C7.1N1 (**C**), GLMP treatment in C14.2N1 (**D**), and GLMP in C17.8N1. (**E**) Blue and purple arrows indicate organic-ammonia GLMP absorption (crater or bowl-shaped depression). Green arrow indicates no GLMP organic-ammonia absorption. Bar (LM) = 150 µm, and bar (VP-SEM) = 10 µm.

**Figure 5:**
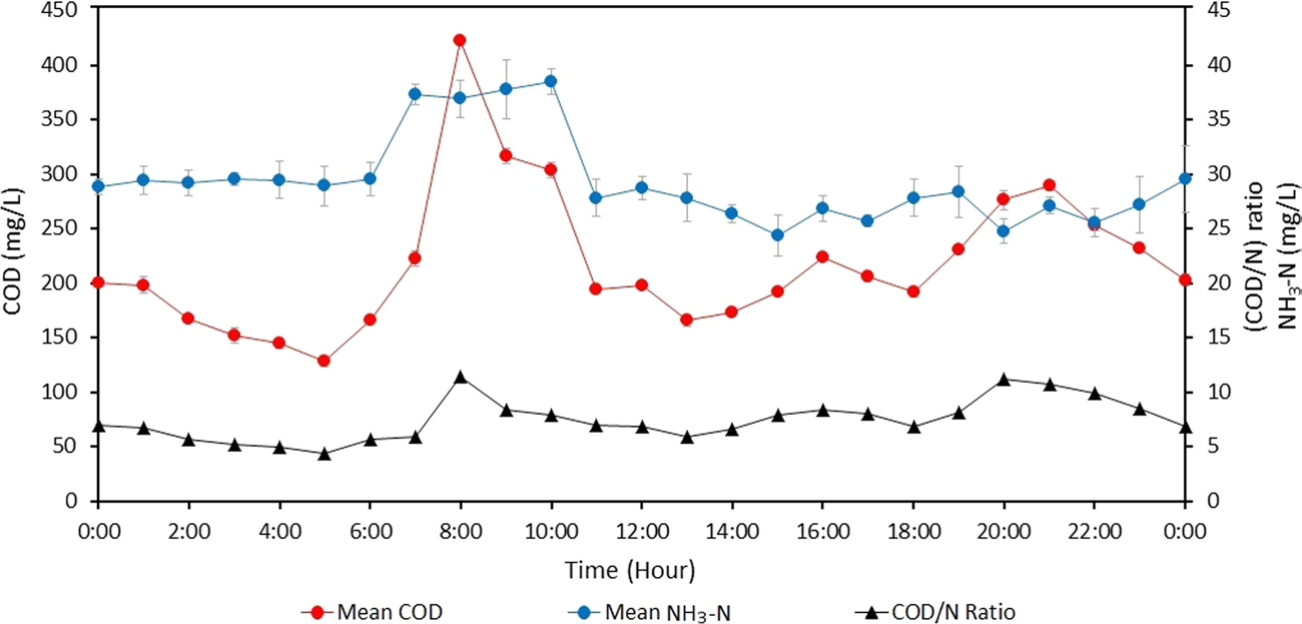
Mean temporal chemical oxygen demand (COD), ammonia nitrogen (NH_3_–N), and carbon to nitrogen (COD/N) ratio trend every 24 hours for three days of sampling of urban domestic Malaysian wastewater as per the inlet basin sewage treatment plant (STP).

**Figure 6:**
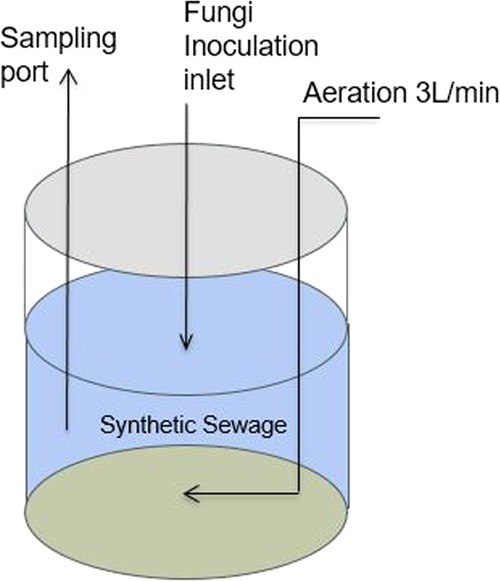
Schematic representation of the batch reactor for lab-scale experimental set-up.

In the current study, the morphological verification of the wastewater–GLMP interaction was performed using a light microscope (LM) and a variable pressure scanning electron microscope (VPSEM) to evaluate the fungal adsorption on the NH_3_–N contaminants (Fig. 4). GLMP showed NH_3_–N absorption in an increasing COD/N gradient manner (C3.6N1; C7.1N1; C14.2N1 and C17.8N1) as compared to the untreated GLMP (transparent mycelial pellet: green arrow: Fig. 4(A)), which corresponded to an increasing grey-pellet coloration (Fig. 4(B)–6(E): purple arrows). In Fig. 4(A), GLMP has an undisrupted (green arrow in LM; smooth pellet surface for VPSEM) spherical shape of the intertwined hyphae (indicating healthy pellets), which is in agreement with the findings of Espinosa-Ortiz *et al*.^34^ and Veiter *et al*.^35^, and the SEM mycelial surface of the medicinal mushroom *Coriolus versicolor*^36^.

Although the GLMP retained its spherical shapes and oval masses after the treatment, changes on the hairy hyphae were much more visible in the LM and in the craters in the VPSEM (indicating unhealthy pellets with longer protuberances), as stated by Wan-Mohtar *et al*.^23^, due to the fungal autolysis possibly indicating NH_3_–N adsorption. For the treated GLMP, the blue arrow (craters) shows the pollutant deposited on the pellet surfaces; this bowl-shaped depression shows the balance residual ammonia deposited on the fungal pellet. There was not much difference in the pellet morphology between the low COD/N and the high COD/N ratios; however, Fig. 4 (C17.8N1) shows the highest grey-pellet coloration. Together, the morphological observations revealed that GLMP successfully adsorbed NH_3_–N in the tested synthetic wastewater.

## Conclusion

The wild-Serbian *G*. *lucidum* strain BGF4A1 (GLSB) had the potential to remove pollutants from the synthetic domestic wastewater. Among all of the tested parameters, the highest carbon to nitrogen (COD/N) ratio showed the best result with a percentage removal of COD and NH_3_–N of 96.0% and 93.2% respectively. This experiment also proved that the fungi worked efficiently under a favourable pH condition, which was in the acidic environment within a pH range of 2.5 to 3.5, consistent with the literature study. The results for the synthetic wastewater revealed the preliminary potential of wild-Serbian GLMPs in treating domestic wastewater, and further studies are necessary on the effect of this fungus on the treatment of real domestic wastewater in order to fully adapt the medicinal mushroom to real-world applications.

## Materials and Methods

### Domestic wastewater characteristics

The temporal characteristics of influent urban Malaysian domestic wastewater into STP with the population equivalent (PE) of 60000 were studied^37,38^. The wastewater was collected using the autosampler ISCO 3700 at intervals of 1 h, where each sampling campaign lasted 24 h. Note that 300 mL of each sample was filled into polyethylene bottles pre-preserved with 0.3 mL of 50% sulphuric acid (H_2_SO_4_)^39^ during each interval and was immediately brought to the laboratory after the sampling collection. The samples were transferred into 50-mL sterile centrifuge tubes, stored in a refrigerator (at 4 °C) until the analysis. The data collection was conducted in the year 2018, and the wastewater was consistently sampled on weekdays between April and August in the inter-monsoon season and at the start of the Northeast monsoon.

Figure 5 shows the mean temporal concentration for COD and NH_3_–N. The data evidently show that the organic content in the incoming wastewater fluctuated over the 24-h period. The COD concentration showed a trend with two distinct peaks where the highest concentration was observed either early morning between 8:00 am to 9:00 am or early night around 8:00 pm. The range of COD concentration was between 117 mg/L and 612 mg/L. The early morning peak had a considerably higher concentration, ranging between 50 and 100 mg/L more than the evening peak. The lowest COD concentration was found late at night between around 0:00 am and 5:00 am. In contrast, when compared with the temporal pattern of the NH_3_–N concentration, the trend for ammonia was somewhat irregular with no common feature observed, except a slightly higher concentration early in the morning around 7:00 am to 10:00 am. The highest and the lowest NH_3_–N concentrations were 49 mg/L and 17.0 mg/L, respectively. The mean temporal concentration for NH_3_–N showed that the concentration did not fluctuate as much as the organic content. Thus, the main parameter that influenced the fluctuating COD/N ratio was the COD concentration.

### Fungal culture and mycelial pellet cultivation

The wild-Serbian medicinal mushroom *Ganoderma lucidum* strain BGF4A1 (GLSB) stock culture was obtained from University of Belgrade, Serbia. The mushroom was isolated from the mountain of Avala (511 m above the sea level) located 16 km southeast of downtown Belgrade City. It was sub-cultured onto a potato dextrose agar (PDA, Sigma-Aldrich, Dorset, UK) slant, incubated at 28 °C for 7 days, and then, stored at 4 °C to avoid any contamination and to maintain its viability. The composition of the medium in grams per litre was as follows: Glucose 10, Yeast Extract (YE) 1, KH_2_PO_4_ (mono-potassium phosphate) 0.5, K_2_HPO_4_ (di-potassium phosphate) 0.5, MgSO_4_ (magnesium sulphate) 0.5, and NH_4_Cl (ammonium chloride) 4, unless otherwise stated. The isolation process was conducted based on the procedure optimised by Hassan *et al*.^19^.

Two seed culture stages were involved in the inoculum preparation of GLSB. Mycelial agar squares (5 mm × 5 mm) were extracted using a sterile scalpel from a 10-day-old plate and inoculated in a 250-mL Erlenmeyer flask containing 100 mL of the optimised medium solution (first seed culture). To produce additional growing hyphae tips, mycelium from the first seed culture were homogenised aseptically by using a hand blender for 10 s. This process was performed under a laminar flow, and then, 20% (20 mL) of the homogenised inoculum for the second seed culture was transferred into a 500-mL Erlenmeyer flask containing 200 mL of the medium. Throughout the process, the inoculum was inoculated into the fresh medium during the late exponential phase (from day 9 to day 11), as the cells were biochemically in their most agile and vital physiological state in this phase. The fungal culture was cultivated for 11 days at 25 °C and 100 rpm with the initial pH of 5, according to the optimum procedures suggested by Lai *et al*.^40^, Supramani *et al*.^21^, and Wan-Mohtar *et al*.^33^.

### Experimental set-up of batch bioreactor for wastewater treatment

Four 1000-mL reactors were filled with 800 mL of synthetic domestic wastewater as per the set-up shown in Fig. 6. The medium of synthetic wastewater was prepared per 1000 mL volume for the following compounds: 100, 200, 400, and 500 mg of glucose, 73 mg of NH_4_Cl (ammonium chloride), 15 mg of Na_2_HPO_4_ (disodium phosphate), 1.5 mg of KH_2_PO_4_ (di-potassium phosphate), 4 mg of CaCl_2_ (calcium chloride), and 2 mg of MgSO_4_.7H_2_O (magnesium sulphate heptahydrate). The same amount of pre-grown wild-Serbian GLMP with an estimated working volume of about 0.1% was inoculated with a sterile pipette into the filled synthetic wastewater at room temperature (approximately 25 °C) in the reactor. The reactor was constantly supplied with 3 L/min of air, creating an aerobic condition and homogenisation to avoid fungal cementation at the reactor wall, because it was run under a static condition with no agitation (Fig. 6). The treatment time for the first cycle was set to 120 h, before being varied for subsequent experiments on the basis of the performance of the organic and ammonia reduction of each cycle.

The experiments consistently used the same synthetic wastewater, prepared according to the modified recipe^15^ to create similar conditions in all the experiments. On the basis of the measured COD shown in Fig. 5, different COD concentrations were prepared to represent the variability in the organic loading, denoted as low (107 mg/L), medium (213 mg/L and 427 mg/L), and high (534 mg/L) with a fixed amount of NH_3_–N of 30 mg/L. The reactors were prepared in duplicates. Theoretically, the COD value corresponds to the concentration of glucose, with 1.067 mg/L COD is equivalent to 1.000 mg/L glucose and the variation of COD allows for a systematic investigation of the influence of COD/N ratios ranging from C3.6N1, C7.1N1, C14.2N1, and C17.8N1. As domestic wastewater is commonly measured at pH 7^41^, whereas fungi are known to be most efficient at pH 4^42,43^, this study investigated the effect of the initial pH of 7, 5, and 4. The pH of wastewater was adjusted by adding 0.1-M HCl to the synthetic wastewater until the desired pH was achieved. Table 1 shows the influent values for all the tested parameters.

**Table 1:** Influent parameters of the synthetic wastewater against chemical oxygen demand to nitrogen (COD/N) ratio synthetic domestic wastewater treatment.

COD/N ratio	COD Influent (mg/L)	NH_3_-N Influent (mg/L)	Initial pH
C3.6N1	107	30	7/5/4
C7.1N1	213	30	7/5/4
C14.2N1	427	30	7/5/4
C17.8N1	534	30	7/5/4

### Sampling and analytical analysis

Throughout the experiment, the daily monitoring values of temperature and pH (using the pH meter, Eutech pH300) were recorded. In the first 24 h of treatment, the wastewater was frequently sampled to investigate the outset reaction of fungi towards the synthetic wastewater as per the set interval specified in Table 2. After 24 h, samples were extracted every 24 h (i.e. on a daily basis). By using a clean pipette, we extracted 15 mL of the liquid during sampling. All the samples were filtered using the Whatman filter paper Cellulose Nitrate Membrane Filters 0.45 µM prior to the analysis to remove the suspended mycelium. The filtered wastewater was then analysed for COD and NH_3_–N. The concentration of COD was estimated by closed reflux, a colorimetric method using HACH vial high-range reactor digestion method (20–1500 mg/L) using the standard protocol from the manufacturer (Method 8000). Meanwhile, NH_3_–N concentrations were measured using HACH nitrogen, ammonia Test ‘N Tube vials salicylate method high range (0.4–50.0 mg/L) using the standard protocol from the manufacturer (Method 10031). Both the methods were viewed using the UV spectrophotometer HACH DR6000. All the analysis was carried out in triplicates and the respective mean ± S.D represented in the graph is shown as error bars. It is noted that those error bars that are not visible in the graph are believed due to their symbols are much smaller than the size of the (data). Once the treatment is completed, the fungi were removed from the system through filtration and became another fungal biomass. The wild-Serbian *G*. *lucidum*, just like any other mushrooms, is the most efficient natural decomposers for organic matter and it can be applied into food and biomass chain^44^. The filtrates can be reusable into value-added biomass and incorporated as rich food-protein resources in landless food production concept^45^ for circular food chains. In addition, fungal biomass has been applied as animal feed ingredients; Shiitake mushroom- Rainbow trout^46^, fingerlings of Carps–Shiitake mushroom^47^, shiitake mushroom–chicken^48^.

**Table 2:** Sampling event frequency for first 24 hours treatment.

Sampling Time (hours)	Interval (hours)
2 (*t*_*2*_)	2
4 (*t*_*4*_)	2
6 (*t*_*6*_)	2
9 (*t*_*9*_)	3
12 (*t*_*12*_)	3
24 (*t*_*24*_)	6

### GLMP observation under scanning electron microscope

The morphology details of the fungi were assessed under a light microscope (Labomed Lx 400) (LABOMED (USA)) connected to a digital camera (iVu 1500) and a Variable Pressure Scanning Electron Microscope (VPSEM) analysis (CARL ZEISS EVO MA 10 (UK) with EDAX APOLLO X (USA)). The fungi before and after treatment with synthetic wastewater were dried in a dehydrator at 35 °C for 2 h to strictly ensure that there was no water in the sample prior to the analysis. The microscopic and VPSEM analysis images for different fungal pellets before and after the treatment with the synthetic wastewater used in the experiments were then compared.

## References

[1] Raboni M, Torretta V, Urbini G (2013). Influence of strong diurnal variations in sewage quality on the performance of biological denitrification in small community wastewater treatment plants (WWTPs). Sustainability.

[2] Ruas G, Serejo ML, Paulo PL, Boncz MÁ (2017). Evaluation of domestic wastewater treatment using microalgal-bacterial processes: effect of CO_2_ addition on pathogen removal. Journal of Apply Phycology.

[3] Cydzik-Kwiatkowska A, Zielińska M (2016). Bacterial communities in full-scale wastewater treatment systems. World Journal of Microbiology and Biotechnology.

[4] Beheshti M, Sægrov S, Ugarelli R (2015). Infiltration / inflow assessment and detection in urban sewer system. Vann Journal.

[5] Pawlowski CW, Rhea L, Shuster WD, Barden G (2014). Some factors affecting inflow and infiltration from residential sources in a core urban area: case study in a Columbus, Ohio, neighborhood. Journal of Hydraulic Engineering.

[6] Ammary BY (2004). Nutrients requirements in biological industrial wastewater treatment. African Journal of Biotechnology.

[7] Mumtaz T (2010). Turning waste to wealth-biodegradable plastics polyhydroxyalkanoates from palm oil mill effluent–a Malaysian perspective. Journal of Cleaner Production.

[8] Mir-Tutusaus JA (2017). Pharmaceuticals removal and microbial community assessment in a continuous fungal treatment of non-sterile real hospital wastewater after a coagulation-flocculation pretreatment. Water Research.

[9] Lucas D (2018). The role of sorption processes in the removal of pharmaceuticals by fungal treatment of wastewater. Science of the Total Environment.

[10] Yesilada O (2010). The evaluation of pre-grown mycelial pellets in decolorization of textile dyes during repeated batch process. World Journal of Microbiology and Biotechnology.

[11] Lucas D, Barceló D, Rodriguez-Mozaz S (2016). Removal of pharmaceuticals from wastewater by fungal treatment and reduction of hazard quotients. Science of The Total Environment.

[12] Zahmatkesh M, Spanjers H, Lier JBV (2018). A novel approach for application of white rot fungi in wastewater treatment under non-sterile conditions: immobilization of fungi on sorghum. Environmental Technolog..

[13] Hai FI, Yamamoto K, Nakajima F, Fukushi K (2008). Removal of structurally different dyes in submerged membrane fungi reactor-Biosorption/PAC-adsorption, membrane retention and biodegradation. Journal of Membrane Science.

[14] del Álamo AC (2017). Removal of pharmaceutical compounds from urban wastewater by an advanced bio-oxidation process based on fungi *Trametes versicolor* immobilized in a continuous RBC system. Environmental Science and Pollution Research.

[15] Xu J, Wang X, Sun S, Zhao Y, Hu C (2017). Effects of influent C/N ratios and treatment technologies on integral biogas upgrading and pollutants removal from synthetic domestic sewage. Scientific Reports.

[16] Ellouze, M. & Sayadi, S. White-rot fungi and their enzymes as a biotechnological tool for xenobiotic bioremediation, 10.5772/64145 Available at, https://www.intechopen.com/books/management-of-hazardous-wastes/white-rot-fungi and-theirenzymes-as-a-biotechnological-tool-for xenobiotic-bioremediation (2016).

[17] Harms H, Schlosser D, Wick LY (2011). Untapped potential: Exploiting fungi in bioremediation of hazardous chemicals. Nature Reviews Microbiology.

[18] Sankaran S (2010). Use of filamentous fungi for wastewater treatment and production of high value fungal byproducts: A review. Critical Reviews in Environmental Science and Technology.

[19] Hassan NA (2019). Efficient biomass-exopolysaccharide production from an identified wild-Serbian *Ganoderma lucidum* strain BGF4A1 mycelium in a controlled submerged fermentation. Biocatalysis and Agricultural Biotechnology.

[20] Nichols N, Dien B, Bothast R (2001). Use of catabolite repression mutants for fermentation of sugar mixtures to ethanol. Applied microbiology and biotechnology.

[21] Supramani S (2019). Optimisation of biomass, exopolysaccharide and intracellular polysaccharide production from the mycelium of an identified *Ganoderma lucidum strain* QRS 5120 using response surface methodology. AIMS Microbiology.

[22] Bouki C, Venieri D, Diamadopoulos E (2013). Detection and fate of antibiotic resistant bacteria in wastewater treatment plants: a review. Ecotoxicology and environmental safety.

[23] Wan-Mohtar WAAQI, Ab Kadir S, Saari N (2016). The morphology of *Ganoderma lucidum* mycelium in a repeated-batch fermentation for exopolysaccharide production. Biotechnology Reports.

[24] Werkneh AA, Rene ER, Lens PNL (2017). Simultaneous removal of selenite and phenol from wastewater in an upflow fungal pellet bioreactor. Journal of Chemical Technolog & Biotechnology.

[25] Mir-Tutusaus JA, Baccar R, Caminal G, Sarrà M (2018). Can white-rot fungi be a real wastewater treatment alternative for organic micropollutants removal? A review. Water Research.

[26] Nair RB, Lennartsson PR, Taherzadeh MJ (2016). Mycelial pellet formation by edible ascomycete filamentous fungi, *Neurospora intermedia*. AMB Express.

[27] Eggert C, Temp U, Eriksson KE (1996). The ligninolytic system of the white rot fungus *Pycnoporus cinnabarinus*: purification and characterization of the laccase. Applied and environmental microbiology.

[28] Birhanli E, Yesilada O (2010). Enhanced production of laccase in repeated-batch cultures of *Funalia trogii* and *Trametes versicolor*. Biochemical Engineering Journal.

[29] Razarinah WARW, Zalina MN, Abdullah N (2014). Treatment of landfill leachate by immobilized *Ganoderma australe* and crude enzyme. ScienceAsia.

[30] Krupa SV (2003). Effects of atmospheric ammonia (NH_3_) on terrestrial vegetation: a review. Environmental pollution.

[31] Revankar MS, Lele SS (2007). Synthetic dye decolorization by white rot fungus, Ganoderma sp. WR-1. Bioresource Technology.

[32] Liu D (2018). Highly efficient removal of ammonia nitrogen from wastewater by dielectrophoresis-enhanced adsorption. PeerJ.

[33] Wan Mohtar WAAQI, Ab. Latif N, Harvey LM, McNeil B (2016). Production of exopolysaccharide by *Ganoderma lucidum* in a repeated-batch fermentation. Biocatalysis and Agricultural Biotechnology.

[34] Espinosa-Ortiz EJ, Rene ER, Pakshirajan K, van Hullebusch ED, Lens PNL (2016). Fungal pelleted reactors in wastewater treatment: Applications and perspectives. Chemical Engineering Journal.

[35] Veiter L, Rajamanickam V, Herwig C (2018). The filamentous fungal pellet-relationship between morphology and productivity. Applied Microbiology and Biotechnology.

[36] Duvnjak D (2016). Advances in batch culture fermented *Coriolus versicolor* medicinal mushroom for the production of antibacterial compounds. Innovative food science & emerging technologies.

[37] Hanafiah ZM (2019). Diversification of temporal sewage loading concentration in tropical climates. IOP Conference Series: Earth and Environmental Science.

[38] Al-Badaii F, Othman MS, Gasim MB (2013). Water quality assessment of the Semenyih River, Selangor, Malaysia. Journal of Chemistry.

[39] Burke PM (2002). Evaluation of preservation methods for nutrient species collected by automatic samplers. Environmental Monitoring and Assessment.

[40] Lai WH (2014). Optimization of submerge culture conditions for the production of mycelial biomass and exopolysaccharides from *Lignosus rhinocerus*. Sains Malaysiana.

[41] Kawan JA, Hassan HA, Suja F, Jaafar O, Abd-Rahman R (2016). A review on sewage treatment and polishing using moving bed bioreactor (MBBR). Journal of Engineering Science and Technology.

[42] Asif, M. B., Hai, F. I., Singh, L. & Price, W. E. Degradation of pharmaceuticals and personal care products by white-rot fungi — a critical review. *Current Pollution Reports*10.1007/s40726-017-0049-5 (2017).

[43] Safri NA, Rafidah J, Kalil MS (2017). Fermentable sugars from agrowastes using cellulase enzyme from local white rot fungi *Pynoporus sanguineus*. Jurnal Kejuruteraan.

[44] Stamets, P. Growing gourmet and medicinal mushrooms, 3rd edn.Chapter 2, p 5–16. Crown Publishing Group, New York (1993).

[45] Rahmann, G., Grimm, D., Kuenz, A. & Hessel, E. Combining land-based organic and landless food production: a concept for a circular and sustainable food chain for Africa in 2100. *Organic Agriculture* 1–13 (2019).

[46] Baba E, Uluköy G, Öntaş C (2015). Effects of feed supplemented with *Lentinula edodes* mushroom extract on the immune response of rainbow trout, *Oncorhynchus mykiss*, and disease resistance against *Lactococcus garvieae*. Aquaculture.

[47] Paripuranam TD, Divya VV, Ulaganathan P, Balamurugan V, Umamaheswari S (2011). Replacing fish meal with earthworm and mushroom meals in practical diets of *Labeo rohita* and *Hemigrammus caudovittatus* fingerlings. Indian J. Anim. Res..

[48] Giannenas I (2011). Performance and antioxidant status of broiler chickens supplemented with dried mushrooms (Agaricus bisporus) in their diet. Poultry science.

